# Prediction of Novel Disease-Related Regions in SIGLEC-7 by *In Silico* and Biochemical Analyses

**DOI:** 10.3390/ijms27125489

**Published:** 2026-06-17

**Authors:** Sayo Morishita, Masaya Hane, Di Wu, Ken Kitajima, Shiho Ohno, Yoshiki Yamaguchi, Chihiro Sato

**Affiliations:** 1 Graduate School of Bioagricultural Sciences, Nagoya University, Nagoya 464-8601, Japan; morishita.sayo.j6@s.mail.nagoya-u.ac.jp (S.M.); hane.masaya.b4@f.mail.nagoya-u.ac.jp (M.H.); wu.di.u5@f.mail.nagoya-u.ac.jp (D.W.); kitajima.ken.k8@f.mail.nagoya-u.ac.jp (K.K.); 2 Integrated Glyco-Biomedical Research Center (iGMED), Institute for Glyco-Core Research (iGCORE), Nagoya University, Nagoya 464-8601, Japan; 3 Division of Structural Biology, Institute of Molecular Biomembrane and Glycobiology, Tohoku Medical and Pharmaceutical University, Sendai 981-8558, Japan; s.ohno@tohoku-mpu.ac.jp (S.O.); yyoshiki@tohoku-mpu.ac.jp (Y.Y.)

**Keywords:** sialic acid, SIGLECs, immune system, pathogenic mutation, InMeRF program

## Abstract

SIGLECs are well-known receptors that distinguish self from non-self by binding to sialic acid-containing glycoconjugates, thereby regulating normal immune functions. They have also been associated with several diseases, including systemic sclerosis, leukemia, and Alzheimer’s disease. To identify pathogenic regions related to ligand binding in SIGLECs using a novel approach, we employed the *in silico* Individual Meta Random Forest (InMeRF) program, which predicts disease-related amino acid substitutions. InMeRF predicted a novel three-amino-acid motif (LSI) consisting of highly pathogenic amino acid residues in SIGLEC-7 and other CD33-related SIGLECs. Alanine substitution experiments and point-mutation energy calculations using SIGLEC-7 as a representative model member of the SIGLEC family showed that mutations in the LSI motif altered binding to ganglioside ligands compared with the wild type (WT) and affected structural stability, as reflected by changes in mutation energy. Structural analysis based on the crystal structure of SIGLEC-7 revealed that the LSI motif forms a buried β-strand located beneath the previously identified sialic acid-binding region (Site 2) in CD33-related SIGLEC-7. Taken together, these findings demonstrate the utility of InMeRF for identifying previously unrecognized pathogenic regions and provide new structural and functional insights into the SIGLEC family.

## 1. Introduction

The immune system is one of the most important biological systems that distinguishes self-cells from non-self-cells. Sialic acid (Sia) is a well-known self-tag used for distinguishing self from non-self via specific molecules such as SIGLECs, also known as Sia-binding immunoglobulin (Ig)-like lectins [1]. Fifteen SIGLECs have been identified in humans, 16 in primates, and nine in mice [2]. These molecules are classified into two groups based on their evolution: classic SIGLECs, whose genes are highly conserved among vertebrates, and non-classical SIGLECs (CD33-related SIGLECs), whose gene similarity is low [2]. To date, 17 crystal structures of SIGLECs have been deposited in the Protein Data Bank, and structural models for all SIGLECs have been predicted using AlphaFold (AF). Structurally, the core architecture of SIGLECs is similar and includes a Sia-binding N-terminal V-set domain, one or more C2-set Ig domains, a transmembrane domain, and a cytosolic region, although the number of C2-set domains varies from 1 to 16 [3].

The V-set domain is the ligand-binding domain responsible for binding Sia-containing glycoconjugates in the immune system [1], whereas the C2-set domain has been considered important for presenting the V-set domain to ligands or for oligomerization [4], although the precise functions of this domain and its ligands remain unclear. The epitope of SIGLECs, Sia, is a group of 9-carbon carboxylated carbohydrates. *N*-acetylneuraminic acid (Neu5Ac), *N*-glycolylneuraminic acid (Neu5Gc), and deaminoneuraminic acid (Kdn; 2-keto-3-deoxy-D-*glycero*-D-*galacto*-nononic acid) are the main forms of Sia; more than 80 structurally distinct Sia molecules are generated through acetylation, sulfation, methylation, lactylation, and lactonization [5,6,7].

In most cases, Sia is present as a monosialyl residue at the non-reducing terminal end of glycans on glycoproteins and glycolipids of mammalian cells and is involved in many biological activities, including roles in the immune system, fertilization, development, and neural functions [5,6,7]. A critical Sia-binding Arg (R) residue is present at Site 1 of the V-set domain, and recently, another Sia-binding region, Site 2, was identified [8,9]. Almost all SIGLECs, except SIGLEC-4, are expressed in immune cells in a cell-type-restricted manner. SIGLEC-4 is present in the brain and peripheral nervous system and is expressed in oligodendrocytes and Schwann cells [10]. Based on their transmembrane and cytosolic regions, SIGLECs are classified into two functional types: inhibitory receptor types, which contain an immunoreceptor tyrosine-based inhibitory motif (ITIM) and/or ITIM-like motifs that regulate inhibitory signaling by recruiting Src homology domain-containing tyrosine phosphatases 1 and 2 (SHP1 and SHP2), and activating receptors, which contain a basic residue in the transmembrane region. Through this region, DNAX-activating protein of 12 kDa (DAP12), an adaptor protein containing an immunoreceptor tyrosine-based activation motif (ITAM), is recruited, thereby activating downstream signaling molecules such as spleen tyrosine kinase (Syk). In addition to mammalian SIGLECs, sialic acid-binding proteins are also found in microbial systems, such as streptococcal Siglec-like adhesins. Structural studies have shown that these adhesins accommodate the entire sialoglycan within a broad binding pocket and engage multiple sugar moieties through extensive interactions [11]. In contrast, although mammalian SIGLECs share a common V-set Ig domain architecture composed of loops and β-sheets, they primarily recognize the terminal sialic acid, highlighting fundamentally different recognition mechanisms [8,9]. These observations underscore the diversity of sialoglycan recognition and provide a broader structural framework for interpreting binding modes such as the involvement of two sites (Site 1 and Site 2) in SIGLECs.

SIGLECs are involved in several diseases, including systemic sclerosis [12], systemic lupus erythematosus (SLE) [13], periodontitis [14], leukemia [15], and Alzheimer’s disease [16]. The relationships between SIGLECs and these diseases have been investigated using genome-wide association studies (GWAS), and associations between SIGLEC genetic polymorphisms and disease susceptibility have been demonstrated [17].

In this study, we attempted to identify the pathogenic amino acids in SIGLECs that may reveal structure–function relationships in lectin–ligand interactions and uncover previously unrecognized functional regions. Recently, a random-forest-based program, Individual Meta Random Forest (InMeRF), was developed to identify disease-related motifs [18]. Using this program, we identified a novel pathogenic motif in polysialyltransferases involved in polysialic acid synthesis and disease pathogenesis [19]. This study demonstrates that, beyond conventionally recognized active sites and surface regions, previously overlooked internal structural support regions are also critical for protein function and disease [19]. Notably, InMeRF enables the identification of such previously unrecognized pathogenic domains.

To gain new insights into disease-related motif structures in SIGLEC-7, we applied Individual Meta Random Forest (InMeRF) to find a novel disease-related motif and confirmed its significance through biochemical and *in silico* analyses.

## 2. Results and Discussion

### 2.1. Identification of Novel Pathogenic Domains in SIGLECs Using the InMeRF Program

**Figure 1:**
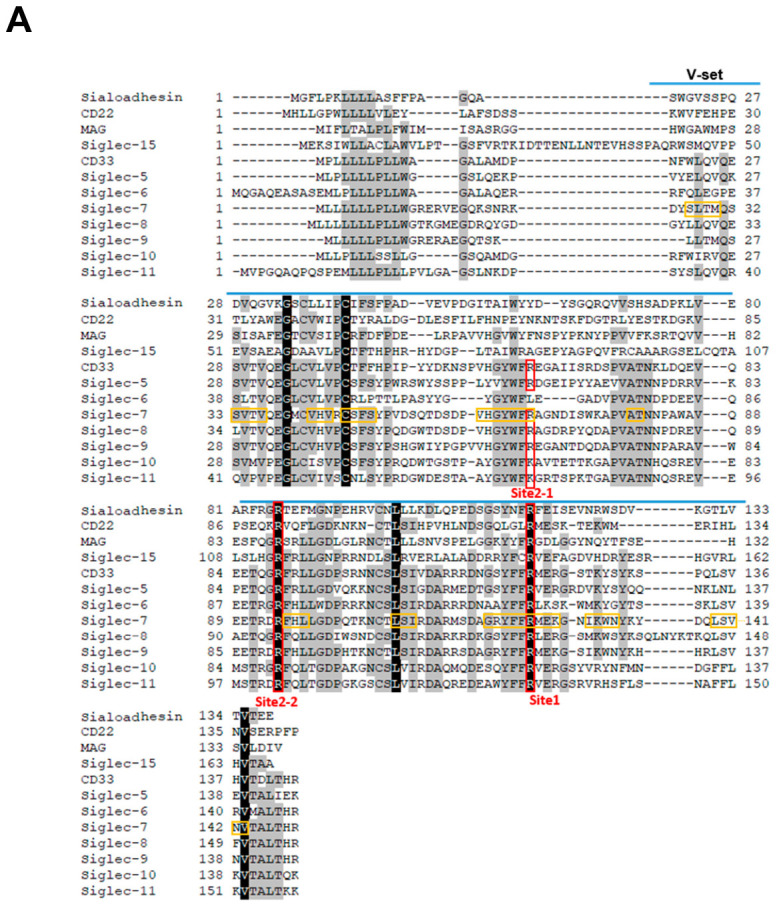

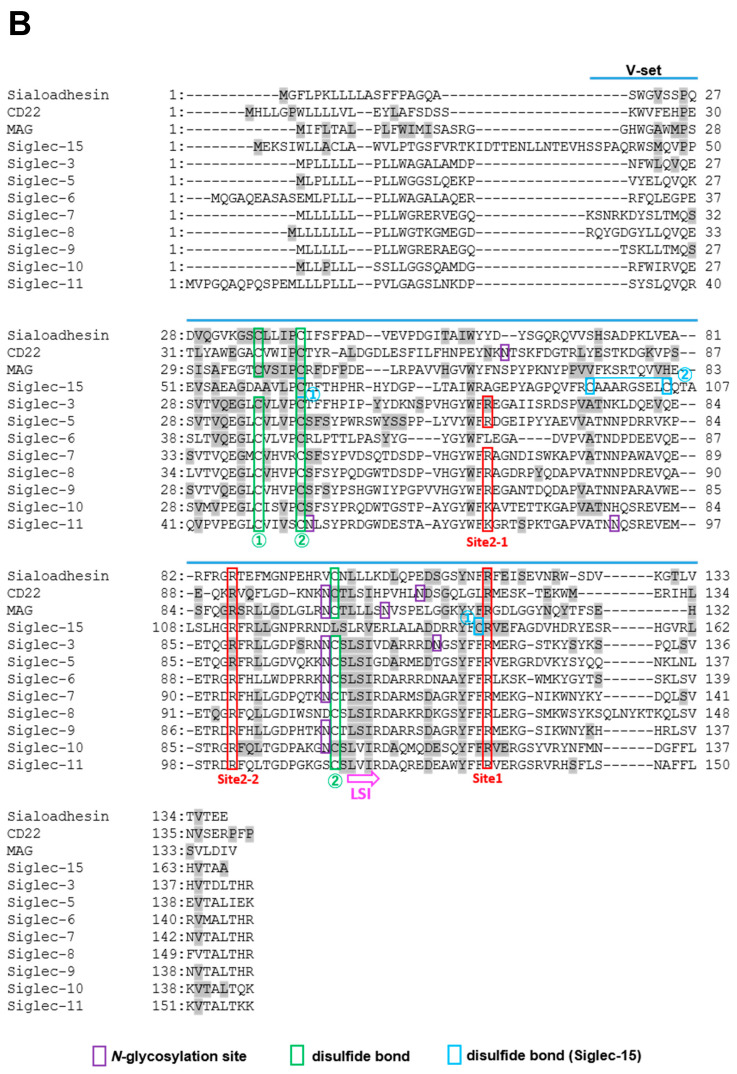

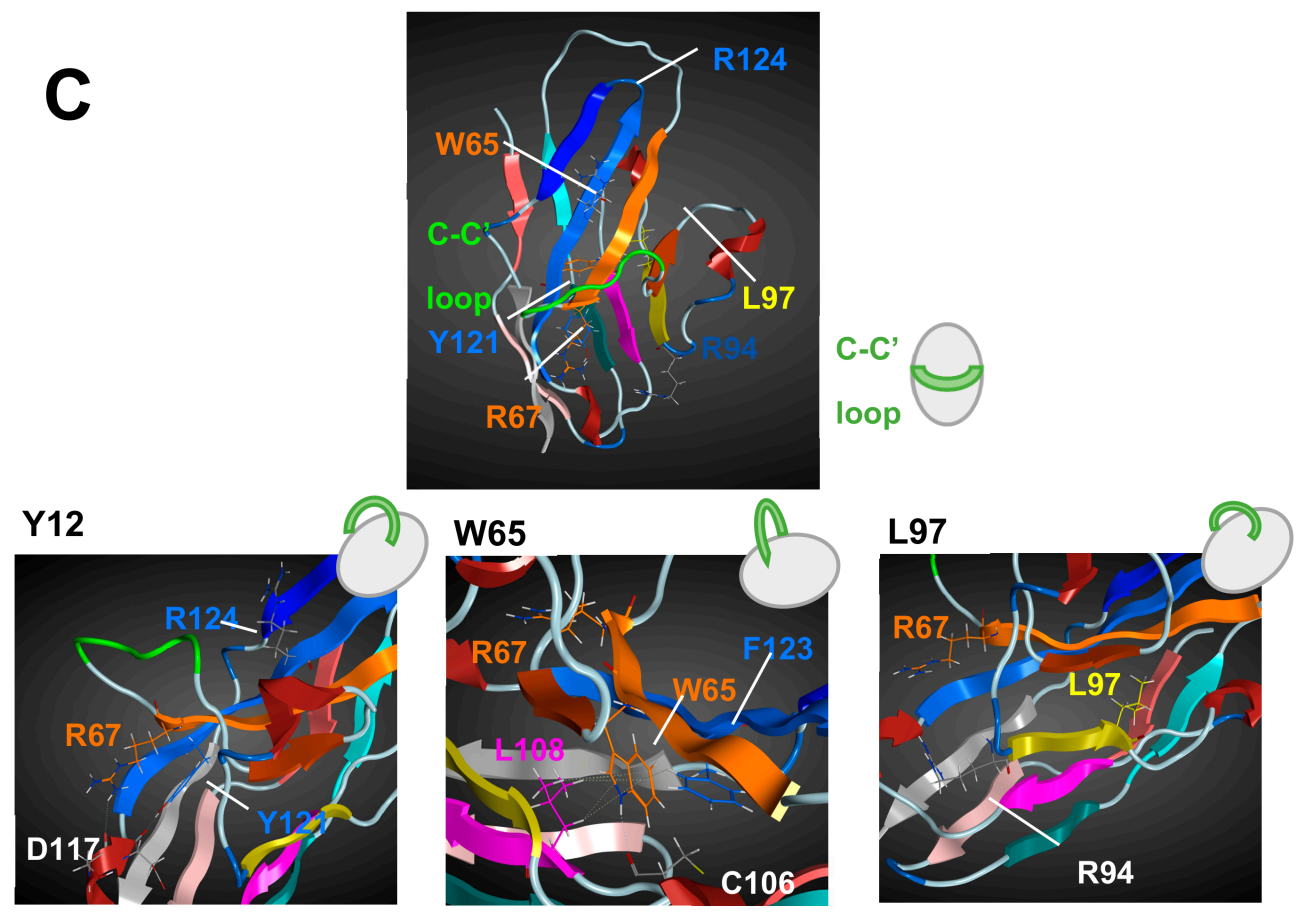
Pathogenicity prediction of non-synonymous single-nucleotide variants using the InMeRF program. (**A**). Alignment of the amino acid sequences of the V-set domains in sialoadhesin (SIGLEC-1), CD22 (SIGLEC-2), MAG (SIGLEC-4), SIGLEC15, CD33 (SIGLEC-3), SIGLEC-5, -6, -7, -8, -9, -10, and -11 using GENETYX version 14. Red and yellow boxes indicate sialic acid-binding sites (R residues) and β-strands, respectively. Fully conserved residues are shown in black, and partially conserved residues are shaded in gray. (**B**). Pathogenicity prediction scores for the V-set domains of the SIGLECs described above. Gray shadow indicates amino acid residues predicted to be pathogenic by InMeRF (>0%). Purple, green, and blue boxes indicate *N*-glycosylation sites, disulfide bonds, and the disulfide bond specific to SIGLEC-15, respectively. Disulfide-bond-forming cysteine pairs are numbered (① and ②). For all SIGLECs except SIGLEC-15, the second cysteine of pair ① is located within the first C2-set domain. Red boxes indicate sialic acid-binding sites. The pink arrow indicates the LSI motif. (**C**). Pathogenic residues Y, W, and L located around Site 1 and Site 2. The leftmost panel shows the crystal structure of the V-set domain of SIGLEC-7 (PDB: 2HRL) as a ribbon model generated using MOE, viewed with the C–C′ loop facing forward. Each β-strand is shown in a different color. The three panels show interactions between amino acid residues predicted to be highly pathogenic and surrounding residues. Cyan indicates hydrogen bonds, and yellow indicates arene-H bonds. The schematic diagram below the panels indicates the orientation of the V-set domain.

First, the amino acid sequences of the V-set domains of SIGLECs were aligned, as shown in Figure 1A. The ligand-binding sites identified by biochemical analyses are indicated as Site 1 (conventional site) [20] and Site 2 (novel binding site) [8,9]. The sequence homology among all SIGLECs, as well as among CD33-related SIGLECs, can be observed. Notably, key basic amino acids in the Sia-binding sites (Site 1, Site 2-1, and 2-2), cysteine residues, and three hydrophobic amino acids are conserved among all SIGLECs or within SIGLEC subgroups (Figure 1A).

To identify pathogenic regions of SIGLECs, we used the InMeRF program, which predicts pathogenic amino acid residues [18]. This program predicts pathogenicity based on random forest (RF) models individually generated for each amino acid substitution to distinguish pathogenic non-synonymous single-nucleotide variant (nsSNVs) in the Human Gene Mutation Database from common nsSNVs in dbSNP [16]. We applied the sequences of the V-set domains of all SIGLECs to this analysis. We omitted the sequences of SIGLEC-14 and SIGLEC-16 because SIGLEC-5 and SIGLEC-11 are paired with SIGLEC-14 and SIGLEC-16, respectively, and their V-set domain sequences are almost identical [21]. In addition, SIGLEC-XII is a pseudo-SIGLEC; therefore, it was omitted from the alignment [2]. The predicted amino acid residues are shown in Figure 1B. These residues are distinct from the homologous residues shown in Figure 1A. Because the amino acids predicted by InMeRF are potentially pathogenic, we sought to characterize the functional importance of these residues through biochemical and *in silico* analyses using SIGLEC-7 as a representative model of CD33-related SIGLECs.

### 2.2. Sialic Acid-Binding Regions in the V-Set Domain

The arginine (R) residue at Site 1 was predicted to be pathogenic mainly in classical SIGLECs, although the R residue at Site 1 is conserved among CD33-related SIGLECs (Figure 1B). This indicates that the R residue at Site 1 is particularly important in classical SIGLECs. A common tyrosine (Y) residue is present on the β-strand containing Site 1, three amino acids before the R residue, and this Y residue was predicted to be pathogenic among all SIGLECs. The Y residue forms a hydrogen bond with an aspartic acid (D) residue, which in turn forms a hydrogen bond with the R residue at Site 2 (Figure 1C, Y121). This suggests that the internal structural support of the R residue may be important for glycan binding at Site 1 across all SIGLECs. Notably, in addition to Site 1, the conventional sialic acid-binding site, Site 2, a previously identified sialic acid-binding site [8,9], was also demonstrated to be functionally relevant in CD33-related SIGLECs.

We then analyzed the Site 2 region, a novel sialic acid-binding site that we previously identified in SIGLEC-7 [8,9]. Only the R residue at Site 2-1 in SIGLEC-3 was predicted to be pathogenic. In the case of Site 2, the tryptophan (W) residue located two amino acids before the basic amino acid on the β-strand containing Site 2-1 was predicted to be pathogenic among SIGLECs, even though the basic amino acid at Site 2-1 is not present in classical SIGLECs. These findings suggest that this site may also function as a structural support region for the presentation of residues important for binding sialoglycoconjugates or other glycoconjugates. The arene–H interactions between W and leucine (L) or between W and phenylalanine (F), together with hydrogen bonds between W and cysteine (C) residues, support this interpretation (Figure 1C, W65). In the case of Site 2-2, the R residues in CD22, MAG, SIGLEC-3, and SIGLEC-5 were predicted to be pathogenic, although all SIGLECs possess the same conserved R residue (Figure 1A). This situation is similar to that at Site 1, because only the R residue at Site 1 in classical SIGLECs was predicted to be pathogenic. In this case, the L residue located three residues after R was also predicted to be pathogenic, suggesting that the region surrounding the basic amino acid, supported by aromatic and/or hydrophobic amino acids, may be important for regulating ligand binding (Figure 1C).

### 2.3. Structural Insight into the LSI Motif in the V-Set Domain

**Figure 2:**
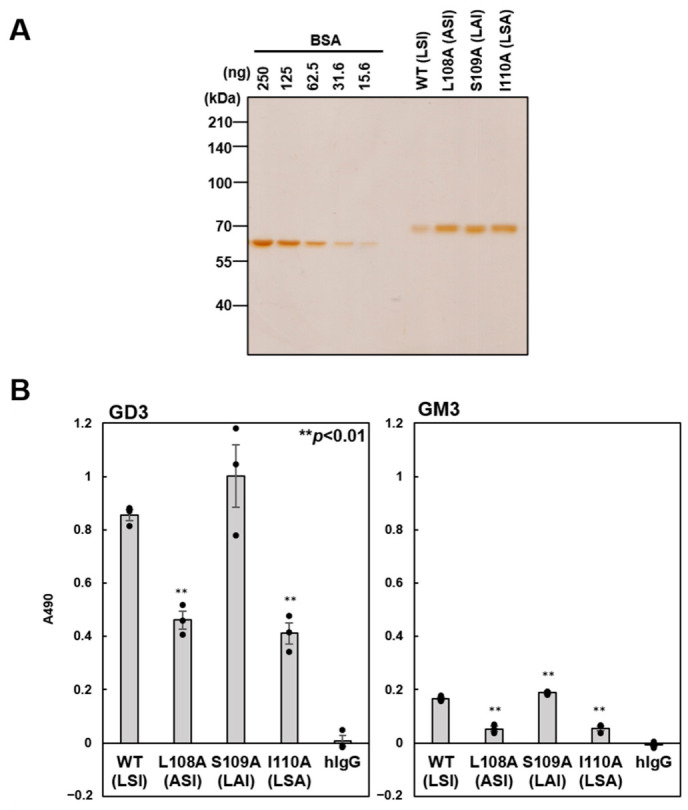
Effects of the LSI motif of SIGLEC-7 on ligand binding. (**A**). Silver staining of LSI and its mutants. Alanine-substitution mutants of the LSI motif (L108A, S109A and I110A) were purified alongside the WT (LSI), and their purity and quantity were confirmed by silver staining. A BSA sample was also loaded as a standard for protein quantification. (**B**). Ligand binding of SIGLEC-7 to GD3 and GM3. Plastic wells were coated with 50 µL of GD3 or GM3 (250 ng/mL) per well. After blocking with PBS containing 0.5% crystallized BSA, the wells were incubated with 50 µL of the Siglec-7-HRP-conjugated anti-human IgG complex. After incubation, color development was performed. Absorbance was measured at 490 nm. Triplicate analyses were performed, and ** indicates *p* < 0.01.

The β-strand consisting of leucine (L), serine (S), and isoleucine (I), designated as the LSI motif (Figure 1B,C and Figure S1), was predicted to be pathogenic in most SIGLECs, although this motif is unlikely to be directly involved in sialic acid recognition and has not previously been a focus of studies on Sia binding (Figure 1B, LSI). We therefore analyzed the relationship between the LSI motif and ligand binding. To clarify whether the LSI motif is involved in Sia binding, plasmids containing alanine substitutions in the LSI motif were prepared and transfected into Expi293 GNT1KO cells. After purification of the LSI mutants (L108A, S109A and I110A), silver staining was performed to confirm the purity and quantify the series of SIGLEC-7-Fc proteins (Figure 2A). We prepared high-quality SIGLEC-7 mutants and analyzed ligand binding toward GD3 and GM3. Although GM3 is known to be a less preferred ligand than GD3, it has been shown to bind SIGLEC-7. Wild-type SIGLEC-7 (LSI) showed strong binding toward GD3 and weak binding toward GM3 (Figure 2B, GD3 and GM3, LSI). The L108A and I110A mutants showed approximately half the binding to GD3 and one-third the binding to GM3 compared with the WT, and these reductions were significant. S109A showed higher binding toward GD3 and GM3 than the WT; however, this difference was not statistically significant in the case of GD3, possibly owing to variability arising from structural instability of the S109A mutant. Overall, these results indicate that amino acid substitutions within the LSI motif altered ligand binding compared with the WT.

**Figure 3:**
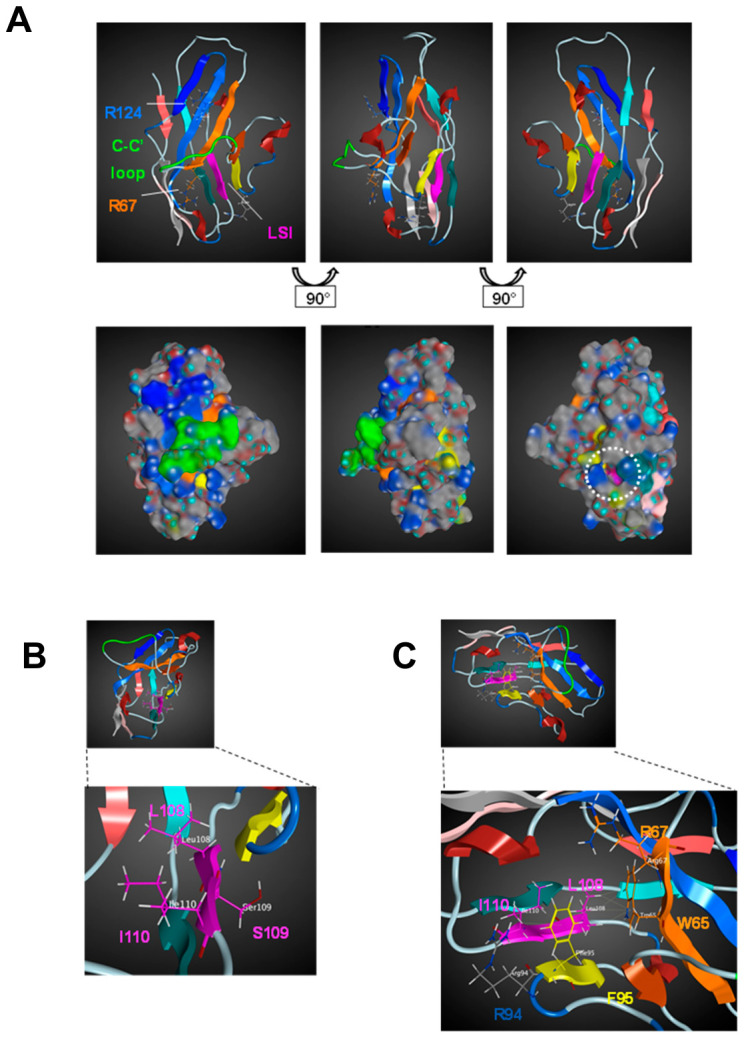
Conformation of SIGLEC-7. (**A**). The structure of the V-set domain of SIGLEC-7 (PDB: 2HRL) is shown as ribbon and space-filling models generated using MOE. The C–C′ loop is shown in green. Residues R124, R67, and R94 correspond to Site 1 and Site 2, respectively, which have been demonstrated to participate in sialic acid binding. The LSI motif, a predicted pathogenic β-strand, is shown in purple; the β-strand containing Site 1 is shown in light blue; and the β-strand containing Site 2-1 is shown in orange. The remaining β-strands are also color-coded. The lower panel shows the surface representation of SIGLEC-7, demonstrating that the LSI motif indicated by a white circle, is buried within the interior of the protein. (**B**). Structure of the LSI motif in SIGLEC-7. (**C**). Interactions between amino acids in the LSI motif and surrounding residues. The β-strand containing F95 is shown in yellow. The arene–H interactions between L108 and W65, and between I110 and F95, are indicated in yellow.

To gain structural insight into ligand binding, we used the crystal structure of SIGLEC-7 (PDB: 2HRL) shown in Figure 3A. SIGLEC-7 consists mainly of two β-sheets composed of multiple β-strands in each domain (Figure 1A and Figure 3A). The LSI motif forms one of these β-strands within the V-set domain (Figure 3A, upper panel, purple ribbon) and is located on the opposite side of the β-sheet cluster from Site 1. Examination of the protein surface of SIGLEC-7 (Figure 3A, lower panel) showed that the main chain of the LSI motif and the side chains of the L and I residues are buried within the molecule. The side chains of L and I are oriented internally, whereas the side chain of S is oriented externally (Figure 3B,C).

Furthermore, interactions were observed between L108 and W65, the latter being predicted to be pathogenic, and between I110 and F95, which was not predicted to be pathogenic but is conserved among CD33-related SIGLECs. W65 is located on the β-strand containing R67 (Site 2-1), whereas F95 is positioned adjacent to R94 (Site 2-2) (Figure 3B,C). These findings suggest that the LSI motif is important for maintaining the Site 2 structure required for Sia recognition. Despite being buried within the molecule, the LSI motif was predicted to be pathogenic. Taken together with the results shown in Figure 2B, the reductions in ligand binding observed for the L108A and I110A mutants suggest that these substitutions disrupt key interactions, preventing the maintenance of a stable Site 2 structure and thereby reducing ligand accessibility to Site 2. In contrast, the S109A mutation may facilitate ligand access to Site 2. However, it is difficult to experimentally demonstrate these mechanisms directly. Both decreases and increases in ligand-binding affinity can be detrimental. Therefore, these findings suggest that InMeRF successfully predicts the pathogenic relevance of the LSI motif. This observation is consistent with our previous findings from a similar analysis of glycosyltransferases [19].

### 2.4. Energy Calculation of Mutations for SIGLEC-7

**Figure 4:**
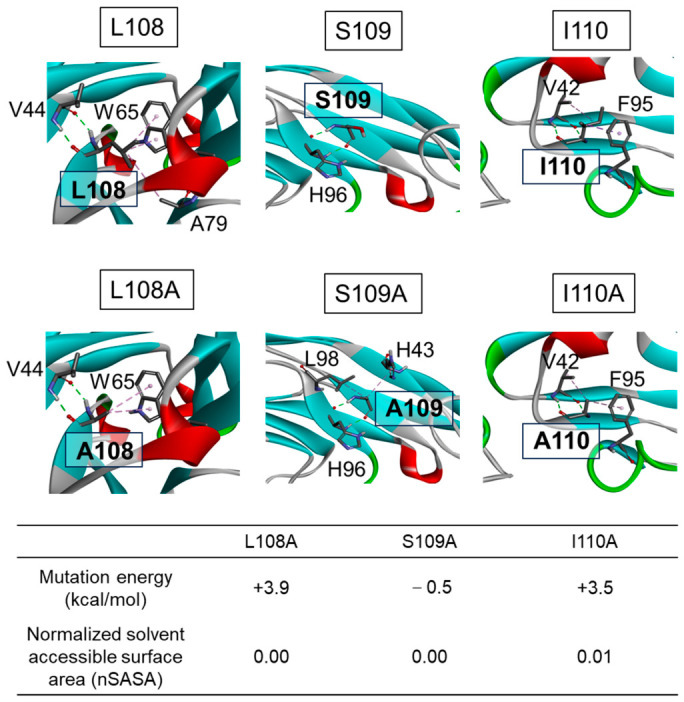
Structural and energetic analysis of the V-set domain of SIGLEC-7, focusing on the LSI motif. Mutation energies (kcal/mol), defined as the differences in Gibbs free energy between the wild type (WT) and alanine mutants (L108A, S109A, and I110A), were calculated. The residues surrounding the LSI motif are shown for both the WT and mutant structures. Hydrogen bonds, alkyl interactions, and π–σ interactions are indicated in green, pink, and purple, respectively. Normalized solvent-accessible surface area (nSASA) values are also shown. All figures were prepared using Discovery Studio 2021.

To evaluate the effects of each alanine mutation in the LSI motif on the conformational stability of SIGLEC-7, we calculated the mutation energy, defined as the difference in Gibbs free energy between the WT and each mutant [19]. The AlphaFold2 model of human SIGLEC-7, which shows high structural similarity to the experimental crystal structure (RMSD = 0.32 Å for Cα atoms; Figure S4) was used for these calculations. The results are shown in Figure 4. Alanine substitutions at L and I (L108A and I110A) exhibited relatively high destabilization energies of 3.9 and 3.5 kcal/mol, respectively. These residues are buried within the protein core, and their substitution is likely to disrupt hydrophobic side chain interactions that contribute to structural stability. Such destabilization is consistent with the reduced ligand-binding activity observed in the biochemical analyses. In contrast, the S-to-A mutation showed only a modest stabilizing effect (–0.5 kcal/mol). Although small, such energetic changes may still influence ligand binding. Previous studies have demonstrated that even minor energetic perturbations can affect protein function [19]. Consistent with this result, the biochemical analysis (Figure 2B) showed a tendency toward slightly enhanced binding to both GD3 and GM3. We previously reported that a change of approximately 1 kcal/mol in critical regions can lead to substantial effects on enzymatic activity [12]. However, mutation-energy calculations primarily reflect the effects of substitutions on overall structural stability and may not fully capture substitution-induced conformational changes, protein dynamics, or other factors contributing to ligand recognition.

Therefore, even subtle energetic differences may contribute to the altered ligand-binding behavior of SIGLEC-7.

### 2.5. InMeRF-Prediction for C2-Set Domains of SIGLECs

We also applied InMeRF to the C2-set domains of all SIGLECs (Supporting Figures S2 and S3). The first C2-set domain of SIGLEC-1, -2, -4, and -15 corresponding all four classical SIGLECs showed a highly pathogenic domain (Supporting Figure S2B), although the amino acid sequences are not homologous among them (Supporting Figure S2A). SIGLEC-10 also showed the same tendency as the three classical SIGLECs, suggesting that the first C2-set domain among SIGLEC-1, -2, -4, and -10 may share common structural roles that have not been previously characterized. In contrast, no such pathogenic region was identified in the second C2-set domain (Supporting Figure S3B). CD33 possesses only two domains in its extracellular regions, a V-set domain and a first C2-set domain, representing the minimal domain architecture among all known SIGLECs, indicating that these two domains are sufficient for function in the immune system. Since the function of the first C2-set domain remains unreported, further investigation is warranted to elucidate its role.

## 3. Materials and Methods

### 3.1. Materials

DNA polymerase KOD Plus Neo was purchased from Toyobo (Osaka, Japan). The PEI Max transfection reagent was purchased from Cosmo Bio (Tokyo, Japan). Polyvinylidene difluoride (PVDF) membranes were purchased from Millipore (Bedford, MA, USA). Skim milk was purchased from the Megmilk Snow Brand (Tokyo, Japan). Goat anti-human IgG horseradish peroxidase (HRP) conjugate was purchased from Jackson ImmunoResearch (West Grove, PA, USA). The enhanced chemiluminescence Western blotting detection reagent (ECL) was purchased from ATTO (Tokyo, Japan). Protein A Sepharose was purchased from Cytiva (Marlborough, MA, USA). The bovine serum albumin (BSA) standard was purchased from Takara Bio (Shiga, Japan). Expi293 GNT1KO cells, Opti-MEM, and Immuno Plate MultiSorp 96-well plates were purchased from Thermo Fisher Scientific (Waltham, MA, USA). Gangliosides GD3 (Neu5Acα2→8Neu5Acα2→3Galβ1→4Glcβ1-Cer) and GM3 (Neu5Acα2→3Galβ1→4Glcβ1-Cer) were purchased from Nagara Science (Gifu, Japan). Crystallized BSA was purchased from FUJIFILM Wako Chemicals (Osaka, Japan). o-Phenylenediamine was purchased from Nacalai Tesque (Kyoto, Japan).

### 3.2. Pathogenicity Prediction of Non-Synonymous Single-Nucleotide Variants by InMeRF

The pathogenicity of non-synonymous single-nucleotide mutations was predicted using InMeRF (InMeRF hg19; available at: https://www.med.nagoya-u.ac.jp accessed on 14 November 2023 or https://github.com/jtakeda-tokai/inmerf_hg19) [18]. The analysis was performed using genome assembly version GRCh37/hg19. Pathogenicity predictions were performed for the following human SIGLEC genes (with Ensembl IDs): *SIGLEC1* (ENSG00000088827), *CD22* (ENSG00000012124), *CD33* (ENSG00000105383), *MAG* (ENSG00000105695), *SIGLEC5* (ENSG00000268500), *SIGLEC6* (ENSG00000105492), *SIGLEC7* (ENSG00000168995), *SIGLEC8* (ENSG00000105366), *SIGLEC9* (ENSG00000129450), *SIGLEC10* (ENSG00000142512), *SIGLEC11* (ENSG00000161640) and *SIGLEC15* (ENSG00000197046).

### 3.3. Comparative Sequence Analysis

All human SIGLEC sequences were obtained from the National Center for Biotechnology Information (NCBI) Gene Database. All sequences were aligned using GENETYX version 14.

### 3.4. In Silico Analysis

The crystal structure of the human SIGLEC-7 V-set domain (PDB ID: 2HRL) was obtained from the Protein Data Bank (PDB, https://www.rcsb.org/ accessed on 15 November 2023), and structural model simulations were performed using Molecular Operating Environment (MOE) 2020 (Chemical Computing Group, https://www.chemcomp.com/). Detailed procedures for the calculation of mutation energy and normalized solvent-accessible surface area are described in Section 3.9. The secondary structures of the V-set domains in SIGLECs, as shown in Supplementary Figure S1, were predicted using a locally implemented version of AlphaFold3 (v3.0.0) and MOE.

### 3.5. Construction of the Plasmids

The plasmid encoding the extracellular domain of wild-type SIGLEC-7 fused to the human IgG1 Fc region and streptavidin-binding peptide (SBP), pBApo-*SIGLEC-7*Ec (WT)-Fc-SBP, was used as a template to prepare LSI mutants. Each amino acid residue in the LSI motif was replaced with alanine. All mutants were produced using KOD Plus Neo according to the manufacturer’s protocol. The oligonucleotide primers used for mutant construction had the following sequences: L108A: Forward: 5′-ACCAAAAATTGCACCgccAGCATCAGAGATGCCAG-3′; Reverse: 5′-GGTGCAATTTTTGGTCTGTGGGTC-3′, S109A: Forward: 5′-ACCAAAAATTGCACCCTGgccATCAGAGATGCCAG-3′; Reverse: 5′-GGTGCAATTTTTGGTCTGTGGGTC-3′, I110A: Forward: 5′-ACCAAAAATTGCACCCTGAGCgccAGAGATGCCAG-3′; Reverse: 5′-GGTGCAATTTTTGGTCTGTGGGTC-3′. To confirm the introduction of mutations, the resulting plasmids were subjected to DNA sequencing at Eurofins Genomics (Tokyo, Japan).

### 3.6. Transfection and Purification of SIGLEC-7-Fc Protein

Expi293 GNT1KO cells (Thermo Fisher Scientific), derived from HEK293 cells with a knockout of *N*-acetylglucosaminyltransferase I (GnTI), were used for SIGLEC-7 protein expression. These cells lack complex *N*-glycans and predominantly produce high-mannose-type glycans (mainly Man5). Expi293 GNT1KO cells (1 × 10^7^ cells) were seeded in 10 cm dishes and cultured for 24 h. The cells were then transfected with 18 µg of each plasmid in 1.2 mL of Opti-MEM containing 48 µg of PEI Max and incubated for 5 d. The culture supernatant was collected, and proteins were purified as previously described [8,9]. As reported previously, the sialic acid on SIGLEC-7 did not influence the sialic acid binding [8].

### 3.7. ELISA Analysis

Immuno Plate MultiSorp 96-well plates were coated with 50 µL/well of GD3 or GM3 (250 ng/mL in ethanol) and incubated for 2 h at 37 °C. The plates were washed with phosphate-buffered saline (PBS) and then blocked with 1% crystallized BSA for 1 h at 37 °C. During blocking, SIGLEC-7-antibody mixtures (0.5 ng/mL SIGLEC-7 and 1:2000 diluted goat anti-human IgG HRP conjugate in 0.1% crystallized BSA) were incubated for 30 min at 37 °C to prepare the SIGLEC-7–antibody complex. After blocking, the plates were washed with PBS. Subsequently, 50 µL of the SIGLEC–antibody complex was added to each well, and the plates were incubated for 2 h at 37 °C. After washing with PBST, 100 µL of o-Phenylenediamine solution (9.2 mM o-Phenylenediamine in 0.1 M Tris-HCl [pH 6.8] containing 0.2% H_2_O_2_) was added to each well. The reaction was stopped by adding 100 µL of 2 M H_2_SO_4_. Absorbance was measured at 490 nm using a Nivo Plate Reader (PerkinElmer, Waltham, MA, USA).

### 3.8. Data Analysis

Data are expressed as the mean ± SD. Statistical analyses were performed using Microsoft Excel (Microsoft Corporation, Redmond, WA, USA). Statistical significance was evaluated using Student’s *t*-test, with *p* < 0.01 considered statistically significant. Data distribution was confirmed to be approximately normal prior to the application of parametric statistical tests.

### 3.9. Calculation of Mutation Energy and Solvent-Accessible Surface Area

The amino acid sequence of human SIGLEC-7 was obtained from the UniProt database (ID: Q9Y286). A three-dimensional structural model of SIGLEC-7 was obtained from the AlphaFold2 Protein Structure Database [22] and a three-dimensional model of each variant was prepared using Discovery Studio 2021 (BIOVIA, Dassault Systèmes, San Diego, CA, USA). Mutation energy was calculated using the Calculate Mutation Energy/Stability module in Discovery Studio 2021 based on the CHARMm (ver. 44.2) force field [23,24]. The solvent-accessible surface area (SASA) of each residue was calculated based on the wild-type protein structure using Discovery Studio 2021. Each SASA was normalized (nSASA) using the reference SASA values [25].

## 4. Conclusions

In conclusion, this study highlights several key findings. First, InMeRF analysis identified novel regions that had not previously been clearly associated with functional significance but may represent pathogenic domains. Second, among these, the LSI motif is buried within the protein structure and, although not directly involved in ligand binding, is suggested to play a structural support role in maintaining the overall conformation. Third, the consistency between the results of the mutational experiments and the energy calculations supports the validity of the structural and computational approaches used in this study. In particular, mutations in the LSI motif appear to influence structural stability and ligand accessibility in the Site 2 region, as supported by both experimental and theoretical analyses. Finally, the present study also highlights the utility of InMeRF for identifying functionally important regions and provides a framework for future investigations of structure–function relationships in other members of the SIGLEC family. As a future perspective, we plan to further analyze the highly pathogenic C2-set domains identified in certain members of the SIGLEC family.

## Data Availability

The data that support the findings of this study are openly available at https://bio.tools/inmerf accessed on 14 November 2023, https://github.com/jtakeda-tokai/inmerf_hg19, or reference number [18].

## Supplementary Materials

The following supporting information can be downloaded at https://www.mdpi.com/article/10.3390/ijms27125489/s1.

## Abbreviations

The following abbreviations are used in this manuscript:

dbSNP	The Single-Nucleotide Polymorphism Database
InMeRF	Individual Meta Random Forest program
nsSNV	Non-synonymous Single-Nucleotide Variant
Sia	Sialic acid
SIGLEC	Sia-binding immunoglobulin (Ig)-like lectin
WT	Wild type
