# Supplementary material for: Prediction of Novel Disease-Related Regions in SIGLEC-7 by In Silico and Biochemical Analyses
Source: Int J Mol Sci. 2026 Jun 17;27(12):5489. doi: 10.3390/ijms27125489 (PMC13299484; doi:10.3390/ijms27125489)
Supplement: Supplementary file 1 [file ijms-27-05489-s001.zip › ijms-4348399-supplementary.pdf]

Supporting Information

Prediction of novel disease-related regions in SIGLEC-7 by *in silico* and biochemical analyses

Sayo Morishita1, Masaya Hane1,2, Di Wu1,2, Ken Kitajima1,2, Shiho Ohno3, Yoshiki Yamaguchi3, and Chihiro Sato1,2*

[1]Graduate School of Bioagricultural Sciences, Nagoya University, Nagoya 464-8601, Japan

[2]Integrated Glyco-Biomedical Research Center (iGMED), Institute for Glyco-core Research (iGCORE), Nagoya University, Nagoya 464-8601, Japan

[3]Division of Structural Biology, Institute of Molecular Biomembrane and Glycobiology, Tohoku Medical and Pharmaceutical University, Sendai 981-8558, Japan

*Correspondence: chi@agr.nagoya-u.ac.jp; +81-52-789-4295

Supporting Figure S1

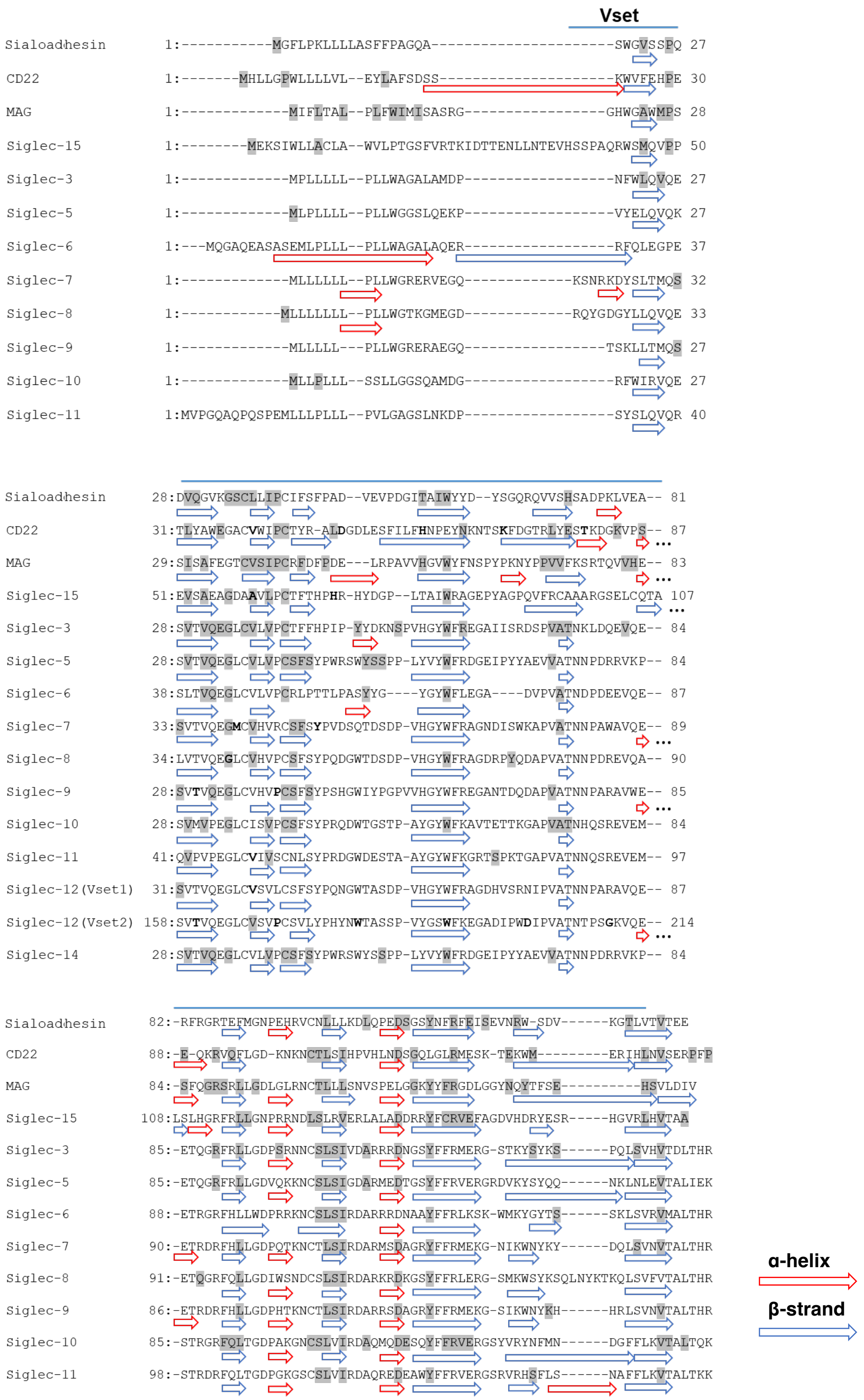

Supporting Figure S1. Secondary structures of V-set domains in SIGLECs. Secondary structures of V-set domains in Sialoadhesin (SIGLEC-1), CD22 (SIGLEC-2), MAG (SIGLEC-4), SIGLEC15, CD33 (SIGLEC-3), SIGLEC-5, -6, -7, -8, -9, -10, and -11. Red arrows indicate α-helix, and blue arrows indicate β-strand. "…" indicates that the α-helix and β-strand are continuous. The Secondary structures were predicted using a locally implemented version of AlphaFold3 and MOE.

Supporting Figure S2

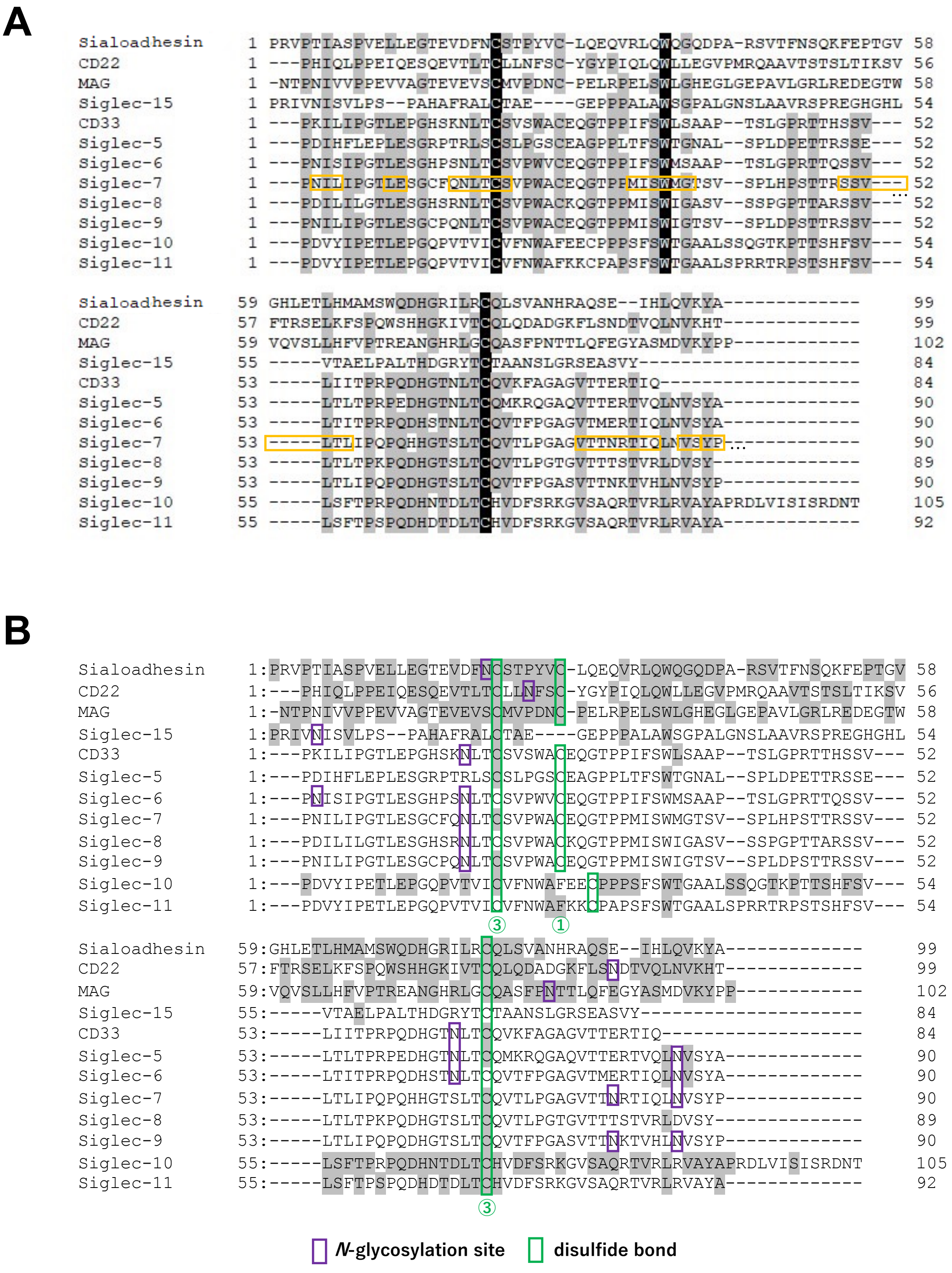

Supporting Figure S2. Pathogenicity prediction in C2-set domain 1 of non-synonymous single-nucleotide variants using the InMeRF program.

A. Alignment of the amino acid sequences of the C2-set domain 1 in sialoadhesin (SIGLEC-1), CD22 (SIGLEC-2), MAG (SIGLEC-4), SIGLEC15, CD33 (SIGLEC-3), SIGLEC-5, -6, -7, -8, -9, -10, and -11. Yellow box indicates β-strands. "…" indicates that the β-strands are continuous.

B. Pathogenicity of the C2-set domain 1 of the SIGLECs described above. Gray indicates amino acid residues predicted to be pathogenic by InMeRF (> 0%). Purple and green boxes indicate *N*-glycosylation sites and disulfide bonds, respectively. Disulfide-bond-forming cysteine pairs are numbered. Except for SIGLEC-15, the other cysteine of pair ① is located within the V-set domain.

Supporting Figure S3

A

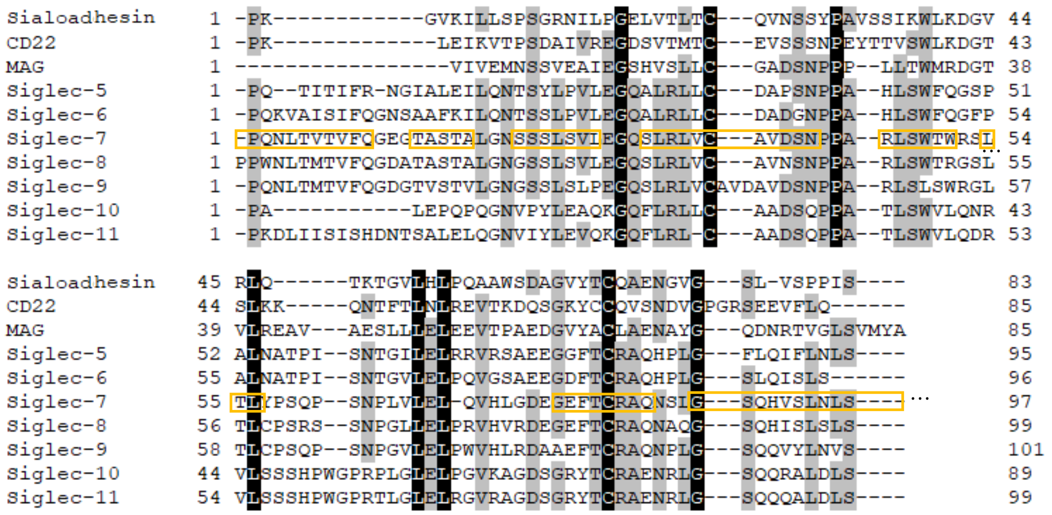

B

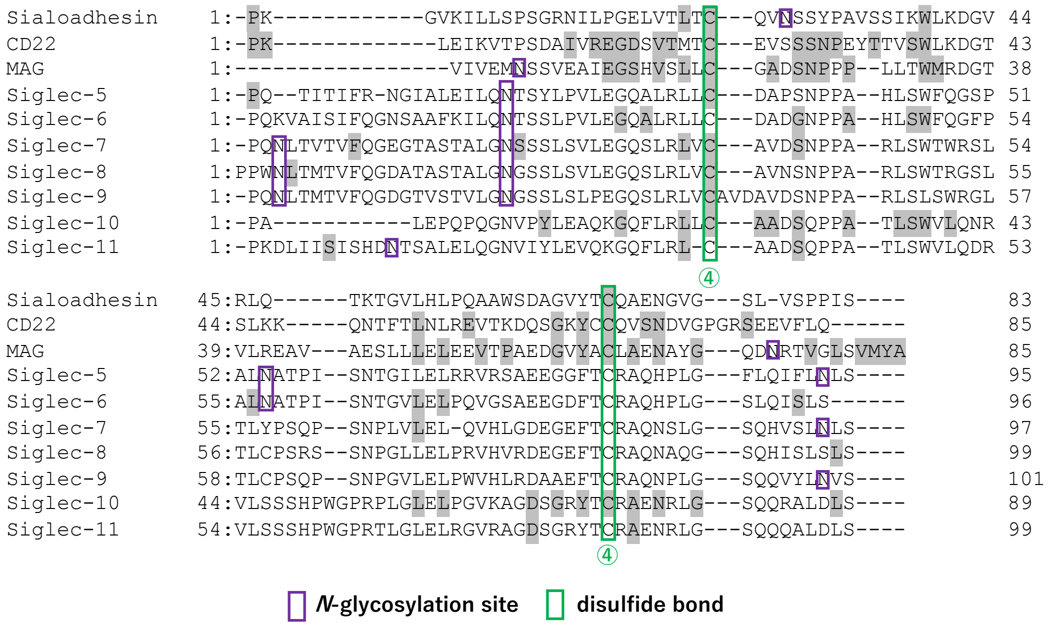

Supporting Figure S3. Pathogenicity prediction in C2-set domain 2 of non-synonymous single-nucleotide variants using the InMeRF program.
A. Alignment of the amino acid sequences of the C2-set domain 2 in sialoadhesin (SIGLEC-1), CD22 (SIGLEC-2), MAG (SIGLEC-4), SIGLEC-5, -6, -7, -8, -9, -10, and -11. CD33 (SIGLEC-3) and SIGLEC-15 were excluded due to the absence of C2-set domain 2. Yellow box indicates β-strands. "…" indicates that the β-strands are continuous.
B. Pathogenicity of the C2-set domain 2 of the SIGLECs described above. Gray indicates amino acid residues predicted to be pathogenic by InMeRF (> 0%). Purple and green boxes indicate *N*-glycosylation sites and disulfide bonds, respectively. Disulfide-bond-forming cysteine pairs are numbered.

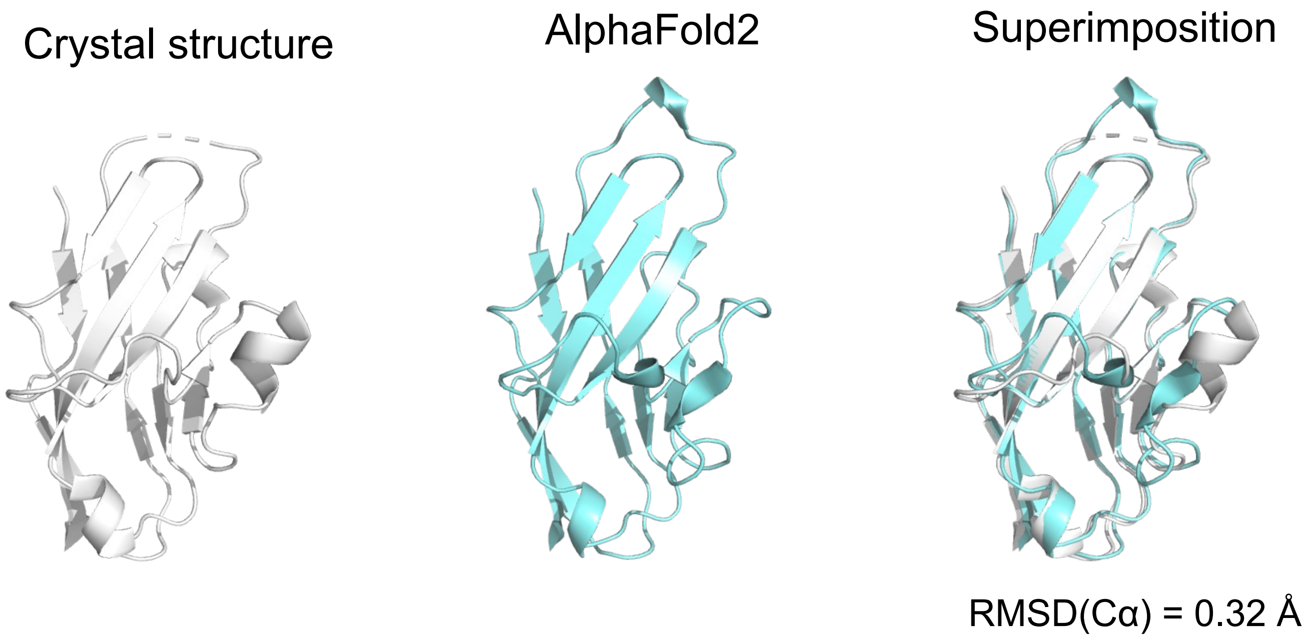

Supporting Figure S4. Comparison of the crystal structure and AlphaFold2 model of the human
SIGLEC-7 V-set domain. A. Crystal structure of the human SIGLEC-7 V-set domain (PDB ID: 2HRL). B. AlphaFold2 model of the human SIGLEC-7 V-set domain. C. Superimposition of the crystal structure (A) and the AlphaFold2 model (B). The root-mean-square deviation RMSD of Cα atoms between the two structures is indicated. All figures were prepared using PyMOL.
